# Sirtuin-2, NAD-Dependent Deacetylase, Is a New Potential Therapeutic Target for HIV-1 Infection and HIV-Related Neurological Dysfunction

**DOI:** 10.1128/jvi.01655-22

**Published:** 2023-01-31

**Authors:** Clara Duran-Castells, Anuska Llano, Ai Kawana-Tachikawa, Anna Prats, Ignacio Martinez-Zalacain, Mie Kobayashi-Ishihara, Bruna Oriol-Tordera, Ruth Peña, Cristina Gálvez, Sandra Silva-Arrieta, Bonaventura Clotet, Eva Riveira-Muñoz, Esther Ballana, Julia G. Prado, Javier Martinez-Picado, Jorge Sanchez, Beatriz Mothe, Dennis Hartigan-O’Connor, Tony Wyss-Coray, Andreas Meyerhans, Magnus Gisslén, Richard W. Price, Carles Soriano-Mas, José Antonio Muñoz-Moreno, Christian Brander, Marta Ruiz-Riol

**Affiliations:** a IrsiCaixa AIDS Research Institute, Hospital Germans Trias i Pujol, Badalona, Spain; b Departament de Biologia, Cel·lular, Fisiologia i d’immunologia, Facultat de Medicina, Universitat Autonoma de Barcelona, Cerdanyola del Valles, Spain; c AIDS Research Center, National Institute of Infectious Diseases, Tokyo, Japan; d Fundació Lluita contra la Sida and Infectious Diseases Department, Hospital Germans Trias i Pujol, Badalona, Spain; e Department of Psychiatry, Bellvitge Biomedical Research Institute-IDIBELL, Barcelona, Spain; f Department of Medicine and Life Sciences (MELIS), Universitat Pompeu Fabra, Barcelona, Spain; g Universitat de Vic - Universitat Central de Catalunya (UVic-UCC), Vic, Spain; h ICREA, Barcelona, Spain; i Centro de Investigaciones Tecnologicas Biomedicas y Medioambientales, CITBM, Lima, Peru; j Department of Medical Microbiology and Immunology, University of California, Davis, California, USA; k Department of Neurology and Neurological Sciences, Stanford University School of Medicine, Stanford, California, USA; l Department of Infectious Diseases, Institute of Biomedicine, Sahlgrenska Academy, University of Gothenburg, Gothenburg, Sweden; m Region Västra Götaland, Sahlgrenska University Hospital, Department of Infectious Diseases, Gothenburg, Sweden; n Department of Neurology, University of California San Francisco, San Francisco, California, USA; o Department of Clinical Sciences, University of Barcelona, Barcelona, Spain; p Department of Psychobiology and Methodology in Health Sciences, Universitat Autònoma de Barcelona, Cerdanyola del Vallès, Spain; q Faculty of Psychology and Education Sciences, Universitat Oberta de Catalunya (UOC), Barcelona, Spain; r Germans Trias i Pujol Research Institute (IGTP), Badalona, Spain; s CIBER Enfermedades Infecciosas (CIBERINFEC), Instituto de Salud Carlos III, Madrid, Spain; Icahn School of Medicine at Mount Sinai

**Keywords:** HIV-1, virus infection control, neurological disorder, plasma proteomics, HIV reservoir, neuroimaging, HIV-associated neurocognitive disorders (HAND)

## Abstract

The implementation and access to combined antiretroviral treatment (cART) have dramatically improved the quality of life of people living with HIV (PLWH). However, some comorbidities, such as neurological disorders associated with HIV infection still represent a serious clinical challenge. Soluble factors in plasma that are associated with control of HIV replication and neurological dysfunction could serve as early biomarkers and as new therapeutic targets for this comorbidity. We used a customized antibody array for determination of blood plasma factors in 40 untreated PLWH with different levels of viremia and found sirtuin-2 (SIRT2), an NAD-dependent deacetylase, to be strongly associated with elevated viral loads and HIV provirus levels, as well as with markers of neurological damage (a-synuclein [SNCA], brain-derived neurotrophic factor [BDNF], microtubule-associated protein tau [MAPT], and neurofilament light protein [NFL]). Also, longitudinal analysis in HIV-infected individuals with immediate (*n* = 9) or delayed initiation (*n* = 10) of cART revealed that after 1 year on cART, SIRT2 plasma levels differed between both groups and correlated inversely with brain orbitofrontal cortex involution. Furthermore, targeting SIRT2 with specific small-molecule inhibitors in *in vitro* systems using J-LAT A2 and primary glial cells led to diminished HIV replication and virus reactivation from latency. Our data thus identify SIRT2 as a novel biomarker of uncontrolled HIV infection, with potential impact on neurological dysfunction and offers a new therapeutic target for HIV treatment and cure.

**IMPORTANCE** Neurocognitive disorders are frequently reported in people living with HIV (PLWH) even with the introduction of combined antiretroviral treatment (cART). To identify biomarkers and potential therapeutic tools to target HIV infection in peripheral blood and in the central nervous system (CNS), plasma proteomics were applied in untreated chronic HIV-infected individuals with different levels of virus control. High plasma levels of sirtuin-2 (SIRT2), an NAD^+^ deacetylase, were detected in uncontrolled HIV infection and were strongly associated with plasma viral load and proviral levels. In parallel, SIRT2 levels in the peripheral blood and CNS were associated with markers of neurological damage and brain involution and were more pronounced in individuals who initiated cART later in infection. *In vitro* infection experiments using specific SIRT2 inhibitors suggest that specific targeting of SIRT2 could offer new therapeutic treatment options for HIV infections and their associated neurological dysfunction.

## INTRODUCTION

The advent of combined antiretroviral treatment (cART) has improved the quality of life of HIV-infected individuals, reducing mortality and the severity of many comorbidities. This is also the case for HIV-associated neurocognitive disorders (HAND), which have been reduced considerably in severity; however, neurocognitive symptoms are frequently found in people living with HIV (PLWH) despite effective cART (1). Despite its incidence, the etiology of HAND is not well understood and may be the consequence of several factors, including non-HIV-associated factors (comorbidities and lifestyle) (2) as well as viral replication, treatment-induced toxicity, and inflammation in the brain (3). In addition, microglial cells and central nervous system (CNS)-resident macrophages have been proposed as potential sites of the viral reservoir in the brain and may be critical for the development of HAND (4). While the most severe form of HIV-related neurological disease, HIV-associated dementia (HAD), has decreased considerably with the advent of cART, the asymptomatic and mild neurocognitive disorders are more frequent and challenging to diagnose (5). In addition, cART-treated PLWH are living longer, and it has been suggested that HIV-accelerated, age-associated cognitive decline increases the number of HIV-infected people affected by neurological disorders (6).

Although various biomarkers have been associated with HAND, such as neopterin (7, 8), there is no clear or specific plasma biomarker to predict, classify, and/or monitor this condition. To define soluble factors in plasma associated with neurological dysfunction and to identify potential new therapeutic targets, we used a customized proteomic array, which was previously employed for the prediction of early onset Alzheimer’s disease (9). The results were validated in a cohort of PLWH that had undergone longitudinal neuropsychological and neuroimaging assessments and started cART either (i) <3 months or (ii) >6 months after the estimated date of HIV acquisition (10). Our results show sirtuin-2 (SIRT2), an NAD-dependent deacetylase, to be correlated with the level of *in vivo* virus control and to be associated with neurological dysfunction. In addition, *in vitro* inhibition studies targeting SIRT2 reduced HIV replication and virus reactivation in different cell types, including T-cell-derived phytohemagglutinin (PHA) blasts but especially in monocyte-derived macrophages and primary glial cells, suggesting involvement of SIRT2 in replication and reactivation of the viral reservoir in the peripheral blood and central nervous system.

## RESULTS

### Sirtuin-2, a novel plasma biomarker in HIV infection.

**FIG 1:**
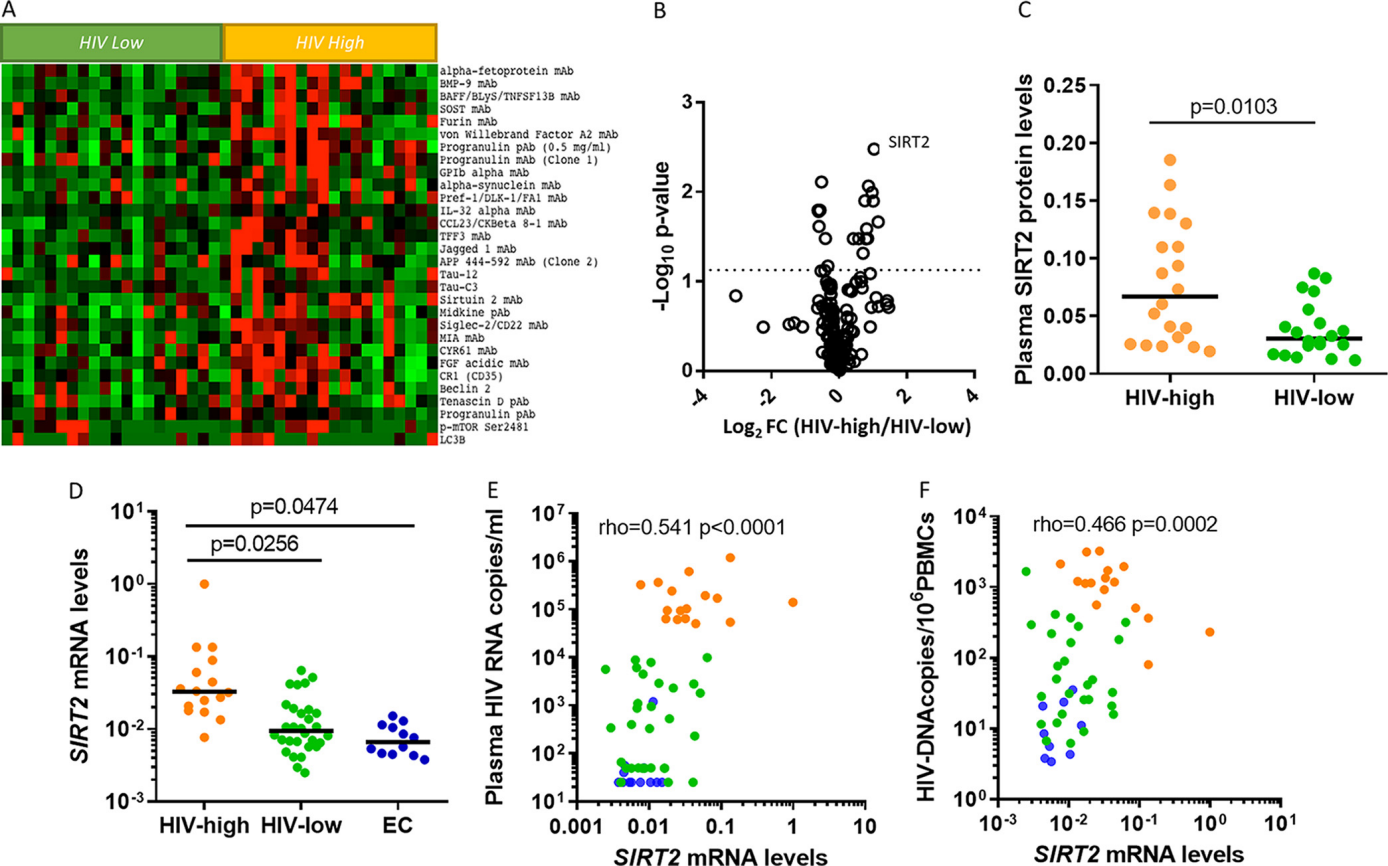
Plasma and gene expression levels of SIRT2 are associated with viral parameters in chronic untreated HIV infection. Proteomic array (9) applied to plasma samples from untreated chronically infected individuals with different degrees of HIV control (*n* = 40). (A) Heatmap showing the relative plasma levels of the most differentially detected soluble factors between untreated HIV-infected individuals with high viral loads (HIV-high, with more than 50,000 viral copies/mL, *n* = 20) and those with low viral loads (HIV-low, viral loads < 10,000 viral copies/mL, *n* = 20). Red indicates high protein abundance in plasma, and green indicates reduced protein levels. (B) Volcano plot representing the relative expression of the 185 molecules measured, identifying SIRT2 as the most significant and differentially detected soluble factor between HIV-high and HIV-low (Log_2_ FC = 1.039; log_10_ *P* value = 2.478). The log_2_ fold change is shown on the *x* axis and the −log_10_ of the *P* value on the *y* axis. (C) Scatterplot showing the relative plasma protein levels of SIRT2 in both study groups (HIV-high [*n* = 20, orange dots] and HIV-low [*n* = 20, green dots]). (D) *SIRT2* gene expression in PBMCs from chronic untreated HIV-infected individuals grouped as HIV-high (*n* = 16, orange dots), HIV-low (*n* = 30, green dots), and elite controllers (i.e., untreated HIV-infected individuals with undetectable HIV viral load in plasma, *n* = 12, blue dots). Study groups are shown on the *x* axis, and relative *SIRT2* gene expression corrected for CD4 counts is shown on the *y* axis. (E and F) Correlation between relative *SIRT2* gene expression and plasma viral load (E) and HIV proviral DNA levels (F) in PBMC for all three groups. HIV-high patients (*n* = 16, orange dots), HIV-low patients (*n* = 30, green dots), and elite controllers (*n* = 12, blue dots) are indicated in the plot. Relative *SIRT2* gene expression corrected for CD4 counts is shown on the *x* axis, and viral load (HIV RNA copies/mL) and proviral levels (HIV DNA copies/10^6^ PBMCs) are shown on the *y* axis. Plasma proteome data were analyzed using the *t* test. SIRT2 plasma levels between HIV-high and HIV-low were analyzed using the Mann-Whitney test. *SIRT2* gene expression levels between HIV-high, HIV-low, and EC were analyzed using ANOVA test for multiple comparisons corrected by original FDR method of Benjamini and Hochberg. The Spearman's rank test was applied for the correlation analysis. Statistical significance was set at *P* < 0.05.

To identify peripheral blood biomarkers associated with the outcome of HIV infection and the development of HIV-related neurological disease, we employed an antibody array previously used successfully in the study of Alzheimer’s disease (9). We measured levels of 185 plasma factors in samples from untreated, chronically HIV-infected individuals classified as “HIV-high” (*n* = 30) with a plasma viral load (pVL) of >10,000 HIV RNA copies/mL (range, 15,000 to 1,200,000; median, 147,500) and “HIV-low” with a plasma viral load of <10,000 HIV RNA copies/mL (range, <50 to 10,000; median, 975) (*n* = 20) (see Table S1 in the supplemental material). Data analyses identified 20 factors as most differentially detected between the two groups (see Table S2 in the supplemental material), of which SIRT2 emerged as the most significant candidate, considering fold change differences of plasma levels and the *P* value of pairwise comparisons (Fig. 1A and B). SIRT2 was elevated in the HIV-high group (Fig. 1C) and the relative plasma levels of SIRT2 correlated positively with pVL and with HIV proviral levels across all HIV-infected individuals studied (Table 1). Correspondingly, the *SIRT2* gene was significantly more highly expressed in peripheral blood mononuclear cells (PBMCs) from the HIV-high (Fig. 1D) than from the HIV-low group. This difference was even more pronounced when comparing the HIV-high individuals to an additional cohort of HIV elite controllers (EC) (HIV-infected individuals with undetectable viral load in the absence of antiretroviral treatment) (Fig. 1D). The same associations with viral parameters observed for SIRT2 plasma levels were also detected in PBMCs, as *SIRT2* gene expression correlated with pVL and HIV proviral levels across all of the groups (Fig. 1E and F).

**TABLE 1:** Top candidates identified by plasma proteomic analysis

Symbol ID	Name ID	*P* value	Fold change	−Log_10_ *P* value^*a*^	Log_2_ fold change^*b*^	VL^*c*^		Proviral^*d*^		CD4 counts^*e*^	
						Rho^*f*^	*P* value	Rho^*f*^	*P* value	Rho^*f*^	*P* value
SIRT2	Sirtuin-2	0.0033	2.0547	2.4785	1.0390	0.441	0.004	0.486	0.034	−0.446	0.001
CCL23	C-C motif chemokine ligand 23	0.0086	1.8287	2.0654	0.8708	0.4470	0.004	0.170	NS^*g*^	−0.321	NS
TFF3	Trefoil factor 3	0.0101	1.9642	1.9940	0.9739	0.526	0.001	0.339	NS	−0.204	NS
AFP	Alpha-fetoprotein	0.0125	1.6965	1.9017	0.7626	0.420	0.007	0.535	0.018	−0.096	NS
GDF2	Growth differentiation factor 2	0.0126	2.0100	1.8993	1.0072	0.345	0.029	0.342	NS	−0.288	NS
GP1BA	Glycoprotein Ib platelet alpha subunit	0.0215	2.2252	1.6670	1.1540	0.429	0.006	0.547	0.015	−0.093	NS
CD22	Siglec-2	0.0258	1.7615	1.5889	0.8168	0.375	0.017	0.355	NS	−0.305	NS
TNFSF13B	TNF superfamily member 13b, BAFF	0.0330	1.7931	1.4809	0.8425	0.392	0.012	0.214	NS	−0.355	0.033
GRN	Progranulin	0.0332	1.3422	1.4795	0.4246	0.336	0.032	0.235	NS	−0.301	NS
VWF1	Von Willebrand factor A2	0.0334	1.7389	1.4763	0.7982	0.305	NS	0.044	NS	−0.241	NS

a Log_10_ *P* value (unpaired *t* test).

b Log_2_ fold change (HIV-high versus HIV-low).

c VL, plasma HIV RNA copies/mL.

d Proviral, HIV-DNA copies/10^6^ PBMCs.

e CD4 counts, cells/mm^3^.

f Rho, Spearman rank test for association analysis performed in HIV individuals (HIV-high and HIV-low).

g NS, not significant.

**FIG 2:**
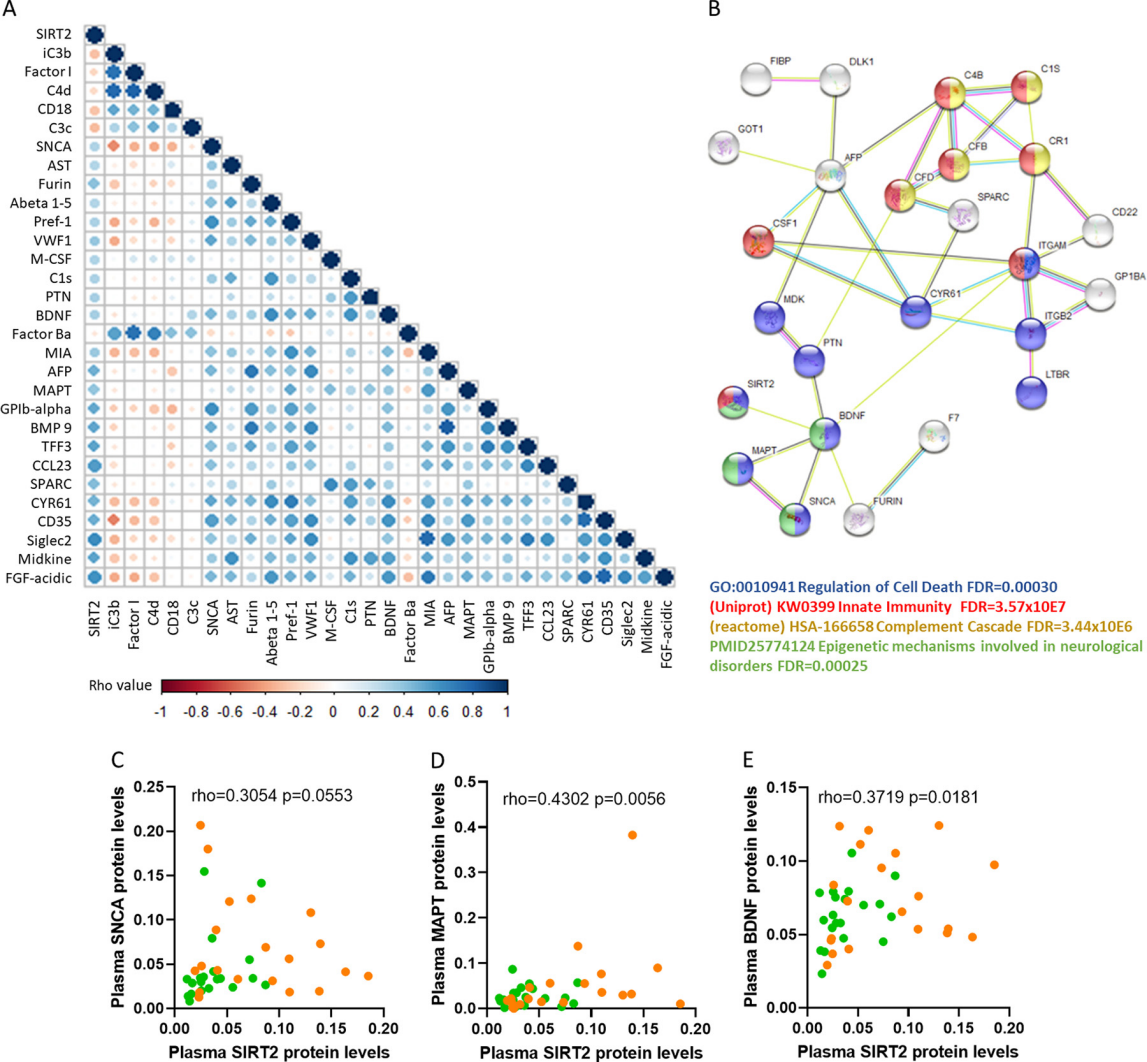
Plasma levels of SIRT2 are associated with neurological factors in chronic untreated HIV infection. (A) Correlation matrix showing the significant relationship between SIRT2 and proteins measured in the antibody array considering all HIV-infected groups (HIV-high and HIV-low). The color scale shows positive correlations in blue and negative correlations in red. (B) The functional analysis performed using the STRING webpage represents the interaction between SIRT2 and the remaining correlated factors. Several functions derived from Gene Ontology, Uniprot, Reactome, and PubMed were identified as follows: *Regulation of Cell Death* category (GO, 0010941; FDR = 0.0030) in blue, *Innate Immunity* (KW0399; FDR = 3.57 × 10E7) in red, *Complement Cascade* (HSA-166658; FDR = 3.44 × 10E6) in yellow, and epigenetic mechanisms involved in neurological disorders (PMID25774124; FDR = 0.00025) in green. (C to E). Correlation plots showing the associations between sirtuin-2 and plasma levels of SNCA (C), MAPT (D), and BDNF (E) measured in the antibody array across all chronic untreated HIV-infected individuals. HIV-high (*n* = 20, orange dots) and HIV-low (*n* = 20, green dots) groups are indicated in the plot. The *x* axis shows relative plasma SIRT2 levels, and the *y* axis shows the relative plasma levels for SNCA, BDNF, and MAPT, respectively. The Spearman's rank test was applied for the correlation analysis. Statistical significance was set at *P* < 0.05.

**FIG 3:**
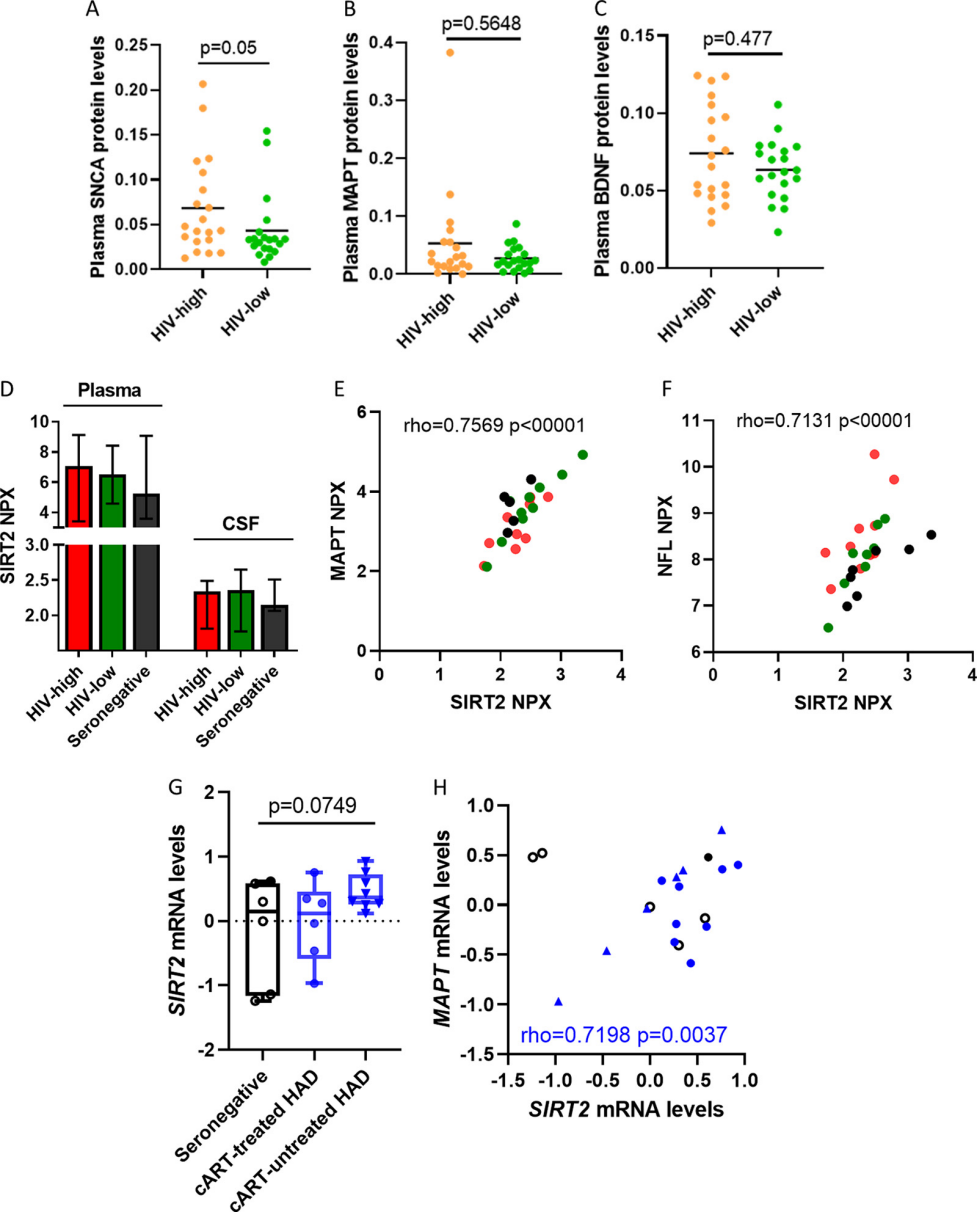
SIRT2 levels in the CNS of HIV-infected individuals. (A to C) Scatterplot showing the relative plasma protein levels of SNCA (A), MAPT (B), and BDNF (C) in both study groups (HIV-high [*n* = 20, orange dots] and HIV-low [*n* = 20, green dots]). (D) Plot representing normalized protein expression (NPX) levels of SIRT2 in the plasma (left) and CSF (right) in HIV-high (*n* = 10, red bar), HIV-low (*n* = 10, green bar), and seronegative individuals (*n* = 5, black bar). The mean and standard deviation are shown. (E and F) Correlation between relative SIRT2 levels and relative CSF levels of MAPT (E) and NFL (F) in HIV-high (*n* = 10, red dots), HIV-low (*n* = 10, green dots), and seronegative individuals (*n* = 5, black dots). Relative MAPT and NFL levels are shown on the *y* axis, and relative SIRT2 levels are shown on the *x* axis. (G) Plot representing *SIRT2* gene expression in postmortem brain tissues measured by microarray in the GSE28160 study performed in seronegative (*n* = 6), cART-treated HAD (HIV-associated disorders, *n* = 6), and cART-untreated HAD individuals (*n* = 8). Values are expressed as mean and standard deviation. (H) Correlation between gene expression levels of *SIRT2* (*x* axis) and *MAPT* (*y* axis) in postmortem brain tissue samples in HIV-infected individuals (cART-treated HAD [*n* = 6] and cART-untreated HAD individuals [*n* = 8]). For comparisons between groups, ANOVA test for multiple comparisons corrected by original FDR method of Benjamini and Hochberg was applied. The Spearman's rank test was applied for the correlation analysis. Statistical significance was set at *P* < 0.05.

In order to study the relationship between SIRT2 plasma levels and the other molecules measured in the array, we performed correlation analysis across all measured markers. Figure 2A shows the molecules in plasma that are significantly correlated with SIRT2 levels (*P* < 0.05, Spearman's rank test). The functional relationship between these statistically related factors was further explored using the STRING application (Fig. 2B). Several functions reported in open-access databases (Gene Ontology, UniProt, and Reactome) were identified, including *Regulation of Cell Death* (GO, 0010941; false-discovery rate (FDR) = 0.00030), *Innate Immunity* (KW0399; FDR = 3.57 × 10^7^), and *Complement Cascade* (HSA-166658; FDR = 3.44 × 10^6^). Interestingly, SIRT2 was functionally connected with brain-derived neurotrophic factor (BDNF), while BDNF was at the same time connected with alpha-synuclein (SNCA) and microtubule-associated protein tau (MAPT) (Fig. 2B), which are both substrates of SIRT2 in brain tissue (11). In addition, SNCA and MAPT are, together with BDNF, well-established biomarkers of neurological damage (12) and correlated positively with SIRT2 levels in plasma (Fig. 2C to E). Although CNS injury was not determined in the two groups of HIV-high/low, trends toward increased levels of specific markers of neurological damage (SNCA, MAPT, and BDNF) in individuals with poor virus control were observed (Fig. 3A to C), with the comparison of SNCA reaching statistical significance. These data identify SIRT2 as a soluble factor in plasma that discriminates between controlled and uncontrolled HIV infection and also showed a relationship with the levels of several biomarkers of neurological damage (13).

To further explore the relationship between SIRT2 levels in plasma and CNS damage in HIV-infected individuals, we determined the levels of SIRT2 in matched cerebrospinal fluid (CSF) and plasma samples from treatment-naive HIV-infected as well as from seronegative individuals. While this small set of samples did not show a significant difference in SIRT2 levels in either group for CSF or plasma (Fig. 3D), the CSF SIRT2 levels correlated strongly with MAPT (*P* < 0.001; rho = 0.7569) (Fig. 3E) and neurofilament light protein (NFL) (*P* < 0.001; rho = 0.7131) (Fig. 3F), a well-known biomarker of neuronal damage in HIV infection (14).

These data were validated by analyzing the open access GEO data set GSE28160, which contains data on postmortem brain tissue from patients that died with diagnosed HAND pathology with a severe symptomatology (HIV-associated dementia [HAD]) (12). We evaluated *SIRT2* expression levels among HIV-infected individuals with cART-treated HAD (*n* = 6) or not receiving cART (cART-untreated HAD, *n* = 8) and seronegative individuals (*n* = 6) (Table 2). As expected from our experimental data, brain *SIRT2* levels tended to be higher in cART-untreated individuals with HAD compared to those of seronegative individuals (Fig. 3G), suggesting increased levels of *SIRT2* in uncontrolled infection. Interestingly, *SIRT2* expression levels in brain tissue from HIV-infected individuals (cART treated and cART untreated with HAD) correlated with *MAPT* expression levels (Fig. 3H), reinforcing the association in our data between SIRT2 and markers of CNS injury.

**TABLE 2:** Clinical information of individuals included in GSE28160 data set

Group^*a*^	Individual^*b*^	Age	Gender^*c*^	Race^*d*^	CD4^*e*^	Plasma vl^*f*^	CSF vl^*f*^	Brain DNA (copies)	Brain RNA (copies)
HIV Seronegative	SN1	44	M	W	NA^*g*^	NA	NA	UN^*h*^	UN
	SN2	30	F	H	NA	NA	NA	UN	UN
	SN3	63	M	H	NA	NA	NA	UN	UN
	SN4	58	F	H	NA	NA	NA	UN	UN
	SN5	57	F	W	NA	NA	NA	UN	UN
	SN6	21	M	H	NA	NA	NA	UN	UN
HAD, cART-untreated	cART-untreated HAD 1	47	M	W	20	210,000	NA	115,468	689,556
	cART-untreated HAD 2	45	F	B	6	NA	NA	58,001	902,832
	cART-untreated HAD 3	44	M	W	7	389,120	>750,000	234,372	762,888
	cART-untreated HAD 4	58	M	B	1	750,000	>750,000	471,248	10,689,754
	cART-untreated HAD 5	43	M	B	10	48,520	134	UN	UN
	cART-untreated HAD 6	33	M	B	1	312,240	<50	l.pos^*i*^	UN
	cART-untreated HAD 7	30	M	B	8	104,300	<50	l.pos	62,826
	cART-untreated HAD 8	44	M	H	16	162,642	NA	UN	702
HAD, cART-treated	cART-treated HAD 1	46	M	W	203	80,000	NA	262,933	2,516
	cART-treated HAD 2	40	M	H	15	750,000	>750,000	3,789	854
	cART-treated HAD 3	51	M	H	136	65	<50	UN	UN
	cART-treated HAD 4	64	F	B	72	359	<50	UN	UN
	cART-treated HAD 5	33	M	W	66	176,800	NA	UN	UN
	cART-treated HAD 6	62	M	W	20	UN	501	UN	UN

a HAD, HIV-associated dementia.

b cART, combined antiretroviral treatment.

c M, male; F, female.

d W, white; H, Hispanic; B, black.

e CD4, number CD4-positive T cells per mm^3^.

f vl, viral load.

g NA, not available.

h UN, undetectable.

i l.pos, low positive by standard PCR.

Overall, these results show that higher levels of SIRT2 in the peripheral blood are detected in uncontrolled HIV infection and that these levels are also correlated with viral parameters as well as with biomarkers of neurological dysfunction.

### Time to cART initiation is associated with plasma SIRT2 levels and neurological dysfunction.

**FIG 4:**
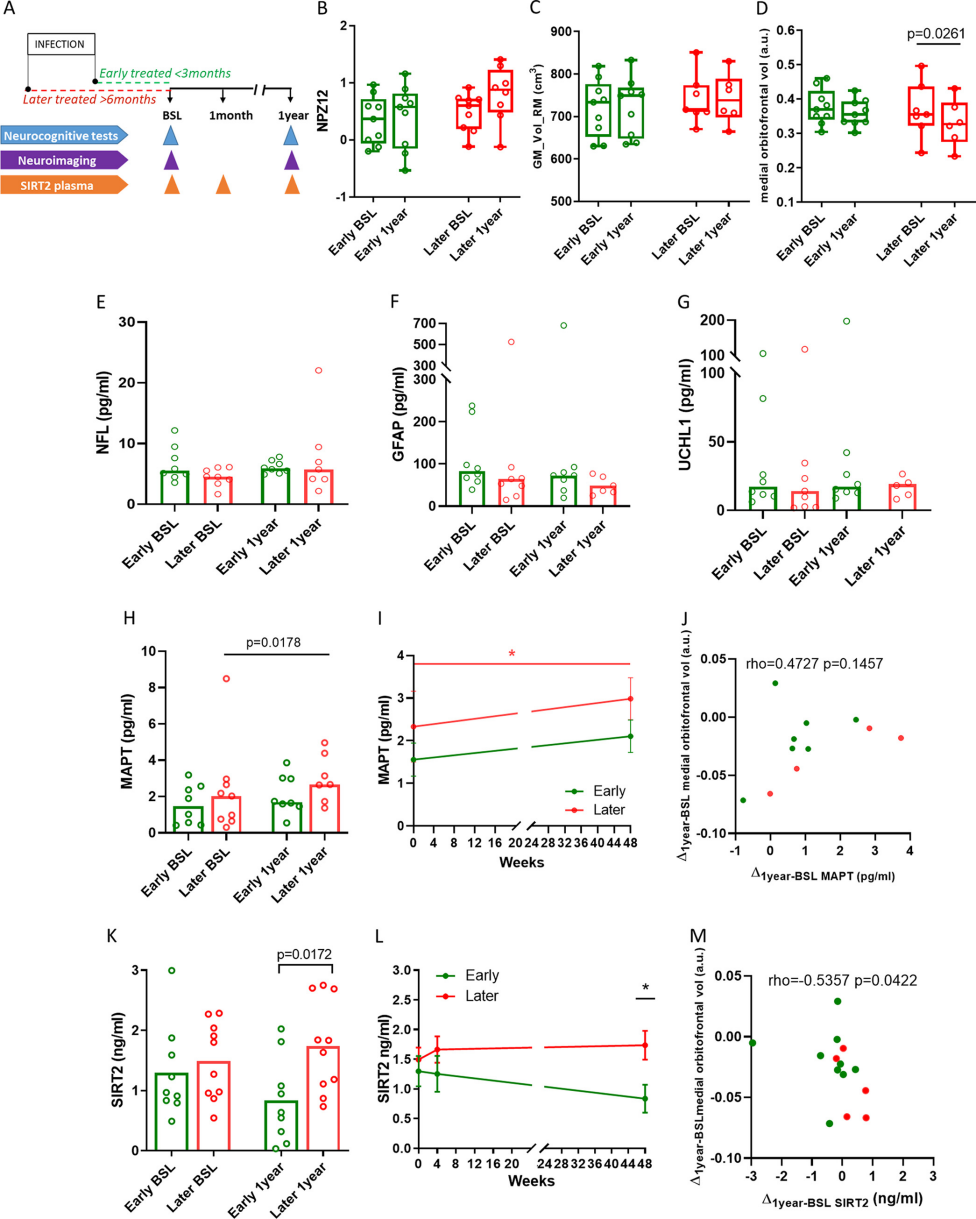
Plasma SIRT2 levels are associated with neurological dysfunction in treated HIV infection. (A) Schematic representation of the following two study arms included in the ARBRE study: early-cART (*n* = 9) and later-cART (*n* = 10) HIV-infected individuals who started cART at different time points after estimated HIV acquisition and underwent longitudinal neurological evaluation (neuropsychological tests and neuroimaging) and quantification of SIRT2 in plasma. Early-cART patients initiated treatment within a maximum of 3 months since the estimated date of infection, and later-treated patients initiated cART at least 6 months after the estimated date of infection. Study visits were at baseline (BSL), 1 month (4 weeks), and 1 year after initiation of cART. (B to D) Plots representing results of the NPZ12 and global gray matter and medial orbitofrontal cortex volume (peak coordinate at MNI, *x* = 6, *y* = 44, *z* = −29) in early- and later-cART individuals at baseline and after 1 year on treatment (early-cART, *n* = 9, green group; later-cART, *n* = 9, red group). The values on the *y* axis represent the NPZ12 score, global gray matter (cm^3^) and medial orbitofrontal cortex volume (a.u.). The box plot shows the median and minimum to maximum values for each group. (E to H) Absolute plasma NFL, GFAP, UCHL1, and MAPT levels at baseline (BSL) and after 1 year on treatment in early-cART (*n* = 9, green group) and later-cART (*n* = 10, red group) individuals expressed in picograms per milliliter. The upper limit of the bar is the median value of protein plasma levels. (I) Absolute MAPT (tau protein) plasma levels represented longitudinally and expressed in picograms per milliliter. (J) Correlation between longitudinal plasma MAPT (tau protein) levels (*x* axis, pg/mL) and the results for medial orbitofrontal cortex volumetry (*y* axis) expressed as the difference between 1 year and baseline in the ARBRE study including early- (*n* = 9, green dots) and later-cART individuals (*n* = 5, red dots). (K and L) Plots representing the absolute plasma SIRT2 in early-cART (*n* = 9, green line) and later-cART (*n* = 10, red line) individuals cross-sectionally (K) and longitudinally (L) at BSL (baseline) and 1 year time points. Weeks of treatment are shown on the *x* axis, and absolute plasma levels of SIRT2 (ng/mL) are shown on the *y* axis. (M) Correlation between longitudinal plasma SIRT2 (*x* axis, ng/mL) and the results for medial orbitofrontal cortex volumetry expressed as the difference between 1 year on cART and baseline in the ARBRE study including early- (*n* = 9, green dots) and later-cART individuals (*n* = 9, red dots). Differences between the groups were analyzed using the Mann-Whitney *U* test, changes over time were assessed using the paired *t* test, and the correlation analysis was based on the Spearman's rank test. Statistical significance was set at *P* < 0.05.

Several studies have suggested that HIV-related neuropathogenesis may be triggered by initial viral entry into the CNS, followed by first pathological processes and the establishment of the viral reservoir in the brain (15). Rapid initiation of cART is currently recommended for all newly diagnosed HIV infections, with expected beneficial effects on brain reservoir and reduced longitudinal neurological dysfunction (16–18). To examine whether the time to cART initiation impacts neurologic status and if SIRT2 plasma levels can be informative of this process, a cohort of HIV-infected individuals who underwent a prospective neurologic evaluation and started cART either (i) <3 months after (“early-cART”) or (ii) >6 months after (“later-cART”) the estimated date of acquisition (Fig. 4A) was studied (8). Both groups were longitudinally evaluated, including baseline (day of cART initiation), 4 weeks, and 1 year, on cART treatment time points (Fig. 4A and Table 3). Plasma viral loads and the estimated days since infection to cART initiation were different between groups at baseline (Table 3). While neuropsychological battery tests (NPZ12 score) and brain global gray matter volume measurements showed no differences between the two groups and over time (Fig. 4B and C and Table 4), an involution of the medial orbitofrontal volumetry was detected in the later-cART after 1 year on treatment (Fig. 4D and Table 4). In parallel, four markers of neurological damage (NFL, glial fibrillary acidic protein [GFAP], ubiquitin C-terminal hydrolase L1 [UCHL-1], and MAPT) were measured in plasma samples (Fig. 4E to H). Only MAPT was increased in the plasma after 1-year on treatment in the later-treated individuals (Fig. 4H and I). Nevertheless, the medial orbitofrontal volumetry and MAPT plasma levels were not significantly associated (Fig. 4J).

**TABLE 3:** Clinical information of neurologically evaluated HIV cohort (ARBRE study)

Parameter	Early cART (*n* = 9)^*a*^	Later cART (*n* = 10)^*a*^
Age, yrs	33 (22–60)	33 (20–48)
Male, *n* (%)	9 (100)	10 (100)
Route of transmission, MSM^*b*^, *n* (%)	9 (100)	10 (100)
Estimated days since infection to cART	70 (12–81)	360 (180–660)
Integrase strand transfer inhibitor (INSTI), *n* (%)		
Raltegravir	2 (22.2)	1 (10)
Elvitegravir	4 (44.4)	3 (30)
Dolutegravir	3 (33.3)	6 (60)
Plasma viral load Log_10_^*c*^	5.3 (3.6–6.7)	4.2 (2–5)
CD4 cell count^*d*^	630 (165–854)	554 (238–936)
CD8 cell count^*d*^	844 (391–1,534)	907 (473–3,662)
CD4/CD8 ratio	0.6 (0.2–1.6)	0.6 (0.2–0.9)
SIRT2 ELISA (*n*)^*e*^	9	10
Cognitive test evaluation (*n*)	9	9
Brain neuroimage assessment (*n*)	9	8

a Values expressed as median with range.

b MSM, men who have sex with men.

c Plasma viral load determined on the day of cART initiation.

d CD4 and CD8 counts are indicated as cells/mm^3^.

e *n*, number of individuals tested by ELISA, cognitive evaluations, and neurological image assessments.

**TABLE 4:** Longitudinal neurological evaluation and SIRT2 plasma levels in ARBRE study

Patients	ELISA SIRT2 (ng/mL)	Global brain image assessment (cm^3^)	Medial orbitofrontal cortex brain image assessment (a.u.)^*b*^	Evaluation of CNS functioning (NPZ12)
Early-cART1_t0	1.875	818,930	0.447	−0.125
Early-cART2_t0	0.796	630,188	0.329	0.842
Early-cART3_t0	1.231	631,082	0.304	0.592
Early-cART4_t0	1.592	760,060	0.382	0.075
Early-cART5_t0	0.49	673,883	0.370	0.967
Early-cART6_t0	0.837	739,790	0.350	−0.200
Early-cART7_t0	0.969	793,197	0.460	0.367
Early-cART8_t0	2.996	697,289	0.401	0.000
Early-cART9_t0	0.912	703,614	0.382	0.575
Early-cART1_t48	1.814	833,048	0.424	−0.533
Early-cART2_t48	0.639	638,473	0.302	0.733
Early-cART3_t48	1.078	634,807	0.333	0.892
Early-cART4_t48	2.026	749,111	0.355	−0.233
Early-cART5_t48	0.319	657,125	0.368	1.158
Early-cART6_t48	0.119	751,895	0.334	−0.075
Early-cART7_t48	0.548	778,229	0.389	0.575
Early-cART8_t48	0.036	705,423	0.396	0.475
Early-cART9_t48	0.946	711,199	0.351	0.642
Later-cART1_t0	0.959	850,686	0.496	0.917
Later-cART2_t0	0.868	753,710	0.366	0.733
Later-cART3_t0	0.547	773,725	0.437	0.708
Later-cART4_t0	2.04	716,441	0.350	0.442
Later-cART5_t0	1.803	670,009	0.243	0.692
Later-cART6_t0	1.909	710,208	0.355	0.158
Later-cART7_t0	1.275	N.D.^*a*^	N.D.	N.D.
Later-cART8_t0	0.979	N.D.	N.D.	0.600
Later-cART9_t0	2.286	711,307	0.322	−0.117
Later-cART10_t0	2.272	N.D.	N.D.	0.217
Later-cART1_t48	1.111	830,125	0.430	1.408
Later-cART2_t48	1.635	762,894	0.322	0.917
Later-cART3_t48	1.187	N.D.	N.D.	0.617
Later-cART4_t48	1.844	713,595	0.332	1.300
Later-cART5_t48	1.836	664,881	0.234	0.833
Later-cART6_t48	2.692	708,699	0.289	−0.125
Later-cART7_t48	0.87	N.D.	N.D.	N.D.
Later-cART8_t48	0.736	N.D.	N.D.	0.992
Later-cART9_t48	2.703	N.D.	N.D.	N.D.
Later-cART10_t48	2.751	774,740	0.376	0.433

a N.D., not determined.

b a.u., arbitrary units.

In addition, SIRT2 plasma levels were similarly elevated after 1 year in the later-cART group compared with those of individuals that initiated cART within less than 3 months from infection (Fig. 4K and L and Table 4). Moreover, SIRT2 plasma levels were negatively correlated with medial orbitofrontal volumetry (Spearman rho = −0.5357; *P* value = 0.0422) (Fig. 4M), indicating that early treatment is crucial for limiting the progressive neurological dysfunction in HIV infection and suggesting that SIRT2 could serve as plasma biomarker for these degenerative processes.

### *In vitro* SIRT2 targeting reduces HIV replication and virus reactivation.

**FIG 5:**
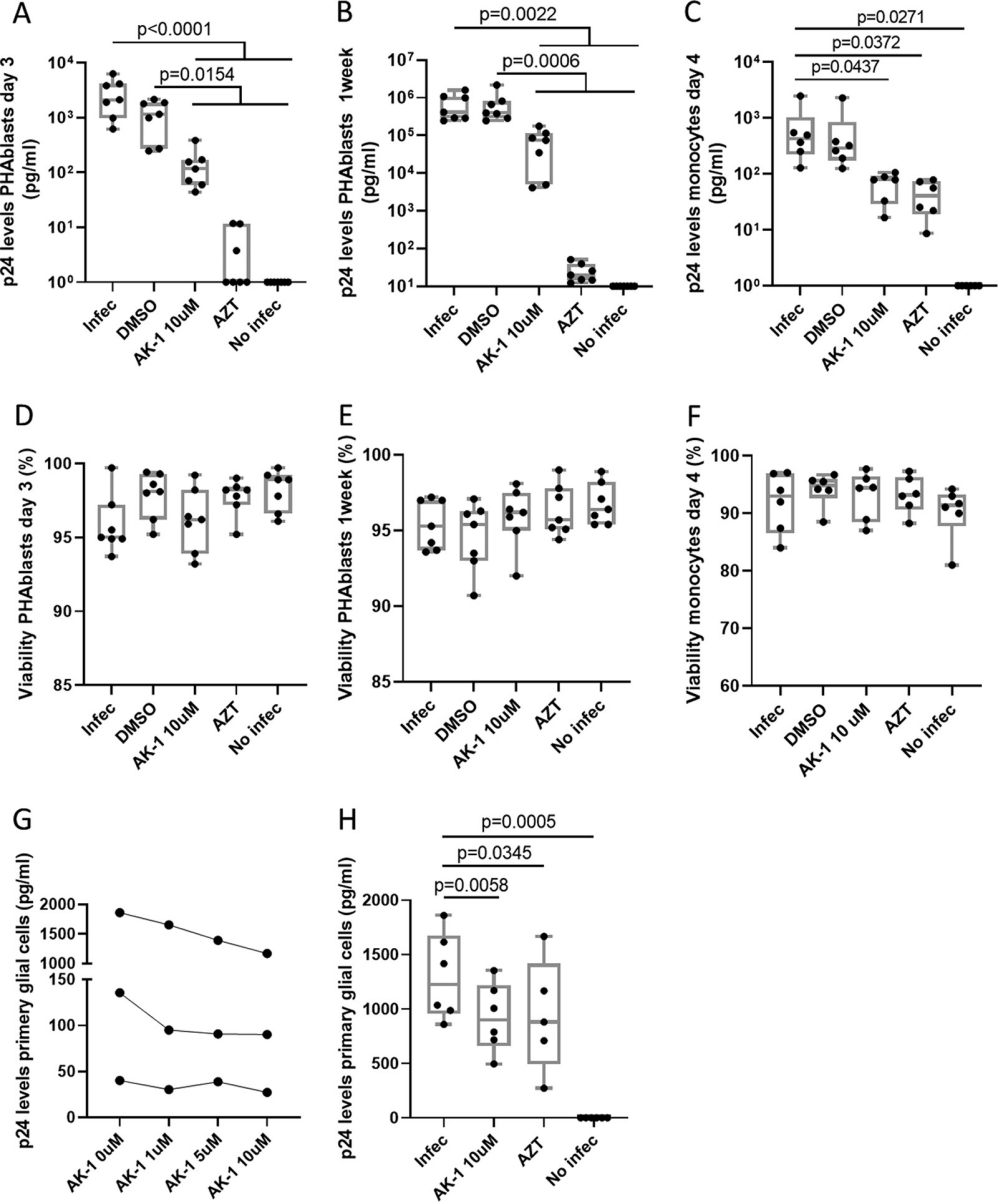
Effect of *in vitro* SIRT2 inhibition on HIV replication. (A to C) Inhibition of HIV replication in the presence of SIRT2 inhibitor (AK-1), tested in HIV-infected PHA blasts (seven independent experiments) (A and B) with HIV_NL4-3_ strain, at day 3 and 1 week postinfection, respectively, and HIV-infected monocyte-derived macrophages (MDMs) (six independent experiments) (C) infected with HIV_BaL_ strain at day 4 postinfection. (D to F) Cell viability in the presence of SIRT2 inhibitor (AK-1), tested in HIV-infected PHA blasts (seven independent experiments) (D and E) with HIV_NL4-3_ strain, at day 3 and 1 week, respectively, and monocyte-derived macrophages (MDMs) (six independent experiments) (F) infected with HIV_BaL_ strain at day 4 postinfection. Experimental conditions are shown on the *x* axis; quantification of viability (% live cells) is shown on the *y* axis. (G) Glial cells infected with the HIV_NLAD8_ virus strain (MOI, 0.01) in the presence of different doses of AK-1 inhibitor. Experimental conditions are shown on the *x* axis; quantification of absolute p24 supernatant (pg/mL) is shown on the *y* axis. The data presented correspond to the mean of two duplicates performed in a single experiment. (H) HIV-infected microglial cells (six independent experiments) with the NLAD8 virus strain in the presence of different doses of AK-1 inhibitor. Experimental conditions are shown on the *x* axis; quantification of p24 levels (pg/mL) is shown on the *y* axis. For results from HIV-infected PHA blasts, MDMs, and primary glial cells, ANOVA test for multiple comparisons corrected by original FDR method of Benjamini and Hochberg was used to analyzed differences between conditions. For all comparisons, *P* < 0.05 was considered significant. The plots show the median of all experiments for each condition.

In order to investigate more directly a potential effect of SIRT2 on HIV replication and virus reactivation, we conducted *in vitro* experiments using specific SIRT2 inhibitors to modulate HIV growth in different target cell types. First, the specific SIRT2 inhibitor AK-1, which is actively being evaluated in Alzheimer’s disease models, was used to inhibit HIV replication in PHA-activated T cells and monocyte-derived macrophages (MDMs) infected *in vitro* with different HIV strains. In the presence of SIRT2 inhibitor, significantly reduced p24 levels were detected in HIV_NL4-3_-infected PHA blasts (at day 3 and after 1 week postinfection) (Fig. 5A and B) and in HIV_BaL_-infected MDMs (day 4 postinfection) (Fig. 5C). The reduction in p24 levels in both cell types was not due to cytotoxic effects, as cell viability was not affected in AK-1-treated compared to uninfected conditions and when comparing PHA-activated T cells to MDMs (Fig. 5D to F). The observed differences of AZT in different cell types are in line with previously published data (19).

As SIRT2 is predominantly expressed in the brain and given its association with markers of neurological damage in the participants of our study (Fig. 2 and 3), we tested its effect on viral replication in primary microglial cells, which are known target cells for HIV and potential sites for brain reservoir (20). Similar to the effects observed in peripheral blood cells, the results from microglial cells showed a significant reduction of HIV replication upon inhibition of SIRT2 (Fig. 5G and H).

**FIG 6:**
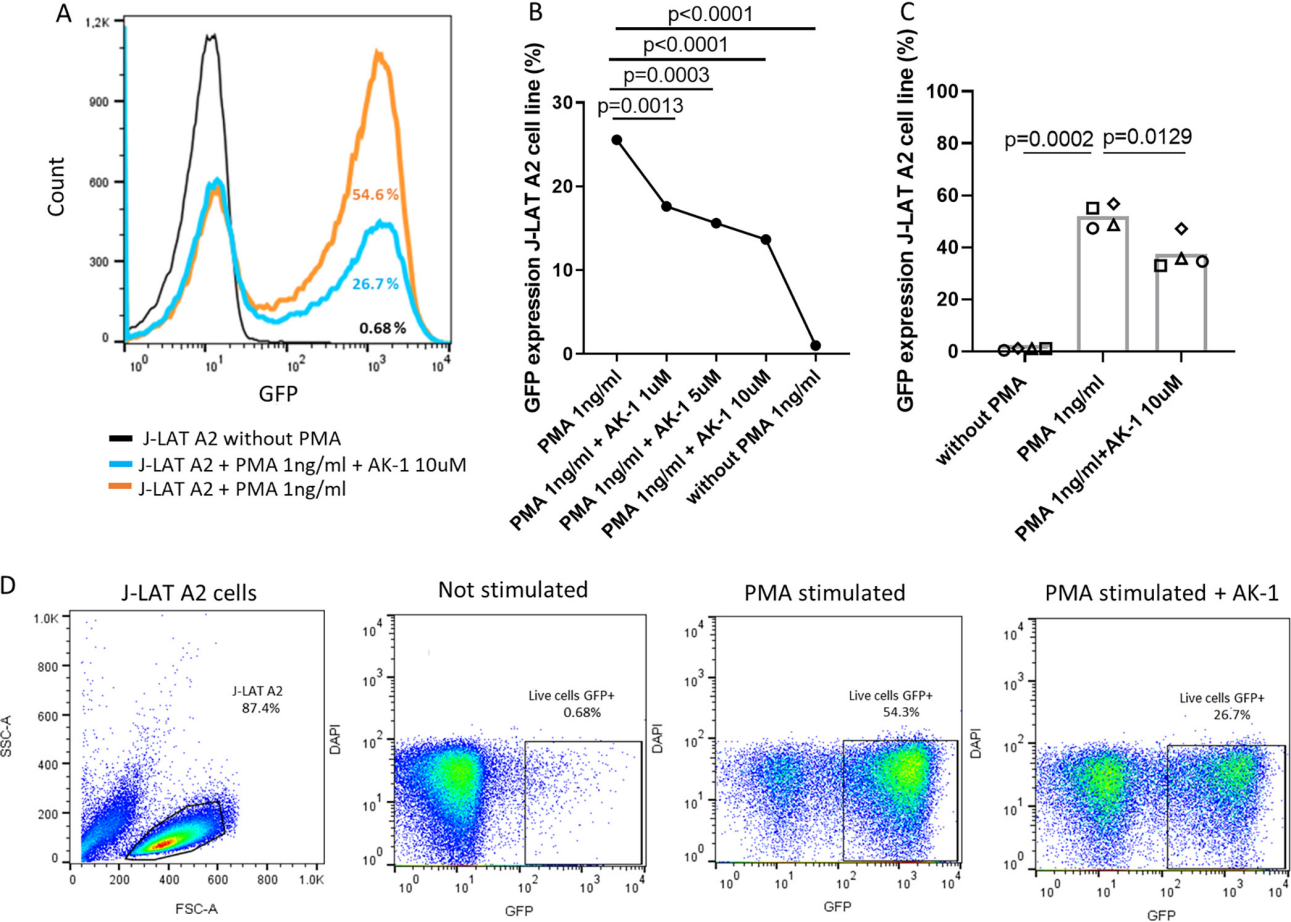
Effect of *in vitro* SIRT2 inhibition on HIV reactivation. (A) Histogram plot showing green fluorescent protein (GFP) cellular counts of three different conditions of the experiment, including nonstimulated cells (black), PMA-stimulated cells (orange), and cells stimulated with PMA in the presence of AK-1 (blue) from one representative experiment. (B and C) HIV reactivation measured by the percentage of GFP expression measured by flow cytometry in J-LAT A2 cells is shown on the *y* axis; different conditions of the experiment, including nonstimulated cells, PMA-stimulated cells, and cells stimulated with PMA in the presence of AK-1 are shown on the *x* axis. (D) Gating strategy of HIV virus reactivation in the J-LAT A2 cell line experiment. For multiple comparisons, ANOVA test corrected by original FDR method of Benjamini and Hochberg was used to analyze differences between conditions. For all comparisons, *P* < 0.05 was considered significant. The plots show the median of all experiments for each condition.

Since SIRT2 plasma and expression levels were also correlated with HIV proviral levels (Table 1 and Fig. 1F), we tested the effects of SIRT2 inhibition on HIV reactivation. Inhibition of SIRT2 with AK-1 significantly reduced reactivation of HIV in the phorbol myristate acetate (PMA)-activated J-LAT A2 cell line (Fig. 6A to C) without affecting cell viability (Fig. 6D). Together, these data suggest that SIRT2 targeting reduces HIV replication and virus reactivation from latency, indicating that SIRT2 is required for effective viral infection in peripheral blood cells and likely in the CNS, given its sites of expression and associations with markers of neurological damage.

## DISCUSSION

Since the advent of cART, the severity of the neurological complications of HIV infection have been drastically reduced (1). However, neurological disorders are frequently reported in PLWH, possibly due to treatment toxicity, inflammation, and viral replication in the brain (7). Factors not directly related to HIV infection, such as comorbidities, coinfections, and lifestyle-related factors, do probably also contribute to cognitive symptom as well as immune activation and other biomarker variations also commonly found in PLWH on suppressive cART (21–23). Some forms of HAND share similar clinical manifestations and underlying mechanisms that may be related to those observed in other neurological diseases, such as Alzheimer’s disease and premature aging (24). However, limited access to CNS tissue complicates the identification of the precise mechanisms involved in the pathogenesis of HAND, hampering early diagnosis of the disease. There is an urgent need to identify plasma biomarkers that are indicative of neurological dysfunction and which could identify novel therapeutic targets in HIV infection. In the present study, we applied an approach that had been previously used for the prediction of the early onset of Alzheimer’s disease (9) to identify plasma factors that are associated with the level of HIV replication and neurological disease.

Using a proteomics platform, we identify SIRT2 (NAD-dependent deacetylase) as the most differentially detected plasma protein between HIV-infected individuals with high and low plasma viral loads. SIRT2 is one of the seven sirtuin family members, which are class III histone deacetylases with a wide range of functions and involvement in multiple processes in the cell (25). Compared to other family members, less research has been carried out for SIRT2. Several target proteins involved in numerous immune and neurological pathways have been identified in recent years, suggesting the involvement of SIRT2 in physiologic and pathological processes. In the context of neurological and inflammatory disorders, a dual effect of SIRT2 in the brain environment has been described, showing that SIRT2 can accelerate the development of neurological pathologies but also protect the brain from deterioration (26, 27). The former aspect has also been described in Alzheimer’s and Parkinson’s diseases, where SIRT2 is thought to contribute to the pathogenic mechanism underlying deacetylation of the α-tubulin molecule and for which specific SIRT2-targeting therapies are under development (28–31).

In our study in chronic HIV infection, we identified a strong association between plasma levels of SIRT2 with other prominent biomarkers of neurological disorders, such as BDNF, MAPT, and SNCA (32–34). These associations are in line with *SIRT2* gene expression being mainly detected in brain tissue (29) and, from our analyses, being produced at especially elevated levels in untreated HIV-infected individuals with HAD (Fig. 3G). Moreover, SIRT2 expression levels in CNS are also associated with MAPT and NFL, further supporting the associations identified at plasma level in HIV infection (Fig. 3).

Past studies have found *SIRT2* to be highly expressed in the temporal cortex of individuals with associated dementia (AD) (29). Our results in PLWH who had undergone longitudinal neuropsychological and neuroimaging assessments also indicate that decreased brain volume near the orbitofrontal cortex was associated with elevated plasma levels of SIRT2. Evidently, more and larger studies determining the *SIRT2* expression patterns in these specific regions of the brain of HIV-infected individuals will be needed to link these observations with neurological outcomes during HIV infection. Still, our data are consistent with findings in a mouse model of frontotemporal dementia, where specific inhibition of SIRT2 by AK-1 in the hippocampus revealed a neuroprotective effect and prevented neuronal loss in this area (33). Indeed, SIRT2 inhibitors have been shown to improve microtubule dynamics and help increase binding of MAPT and SNCA to α-tubulin (28). As such, AK-1 and other SIRT2 inhibitors are being tested in *in vitro* and *in vivo* models of Parkinson and Alzheimer disease (23, 26).

In addition, the role of SIRT2 in infections has recently begun to be explored (27). Specifically, SIRT2 accelerates viral replication of hepatitis B virus (HBV), and the use of sirtuin inhibitors has been proposed as potential new therapeutic interventions (35, 36). In Listeria monocytogenes infection, SIRT2 translocates to the nucleus and deacetylates H3K18, which associates with a subset of host genes that are crucial during the bacterial life cycle (37). Furthermore, Helicobacter pylori infection upregulates *SIRT2* expression in gastric epithelial cells, and specific inhibition is being considered as a therapeutic opportunity (34). Similarly, in chronic Staphylococcus aureus infection in mice, the survival rate was increased with SIRT2 deficiency (38), and in an SIRT2^−/−^ murine model, bacterial infections were reduced (37). More recently, in the context of HIV infection, the potential roles of SIRT2 in some HIV-associated comorbidities (insulin resistance and cardiovascular diseases), but also with neurocognitive disorders (39) and virus life cycle (40), have emerged. In particular, SIRT1, SIRT2, and SIRT3 can deacetylate and regulate Tat activity and, specifically for SIRT1, the interaction with Tat protein was shown to activate the HIV promoter (41). Similarly, with these observations, the present study shows that natural control of HIV infection in the absence of cART is associated with lower SIRT2 levels and that plasma protein and gene expression levels correlate positively with pVL and HIV proviral levels. Our results also show that *in vitro* inhibition of SIRT2 activity by AK-1 in HIV-infected PHA blasts and in MDMs reduced HIV replication, suggesting that HIV (as other pathogens) may have evolved to hijack sirtuins to enhance their replication (42).

It is widely accepted that early initiation of treatment is crucial for reducing the size of the HIV reservoir in different anatomical compartments, including the CNS (18, 43). Particularly, frontal white matter seems to be the main site of HIV reservoir compared to other cerebral regions (44), with microglial cells and macrophages being major compartments harboring HIV-DNA. Interestingly, a number of studies have addressed the biological actions of SIRT2 on microglial cells and macrophages, all outside of HIV infection. In a murine model for neurological inflammation, SIRT2 was shown to drive brain injury and activation of microglia upon stimulation with lipopolysaccharides (45, 46). The results that we obtained after *in vitro* infection of glial cells directly support this model and suggest that SIRT2 plays an important role in brain injury and the HIV viral cycle, including maintaining the HIV reservoir in the brain.

Limitations of this work are the relatively small study size, availability of samples, and limited neurological evaluation time points, as well as age and sex bias in some of the analyses. While we have not observed any association between SIRT2 plasma levels and age in the studied cohort, the sex bias, especially in early HIV infection, is a recurrent limitation in the field, and more female-centered studies are urgently needed to overcome this gap. Despite these limitations, the present study is the first to link SIRT2 levels to the pathological neurological process in HIV infection and an important role in the HIV life cycle and viral reservoir. These results offer new prospect for the development of therapeutic interventions aiming at HIV cure and restoration of neurological dysfunction.

## MATERIALS AND METHODS

### Patients.

Chronic untreated HIV-infected individuals (*n* = 60) enrolled at the IMPACTA clinics (Peru), Hospital Germans Trias i Pujol (Spain), Sahlgrenska University Hospital in Gothenburg (Sweden), and University of California San Francisco (USA) were classified according to their degree of control of viral replication (see Table S1 in the supplemental material). HIV-infected participants from the ARBRE study (ClinicalTrials registration no. NCT03835546) recruited at Fundació Lluita per la Sida, Hospital Universitari Germans Trias i Pujol, (Spain) who underwent longitudinal neuropsychological and neuroimaging assessments (Tables 2 and 3), were also included. These participants were divided into 2 arms according to the time from estimated date of HIV acquisition to initiation of cART. The “early-cART” arm (*n* = 9) started cART within less than 90 days and the “later-cART” arm (*n* = 10) longer than 6 months since estimated time of HIV acquisition (Tables 2 and 3) (8). Available plasma, CSF, and dry-pellet PBMC were stored until use. Blood samples from non-HIV-infected donors from the Banc de Sang i Teixits in Barcelona for *in vitro* studies were used. The study was approved by the Comité Ètic d’Investigació Clínica of Hospital Germans Trias i Pujol (CEIC EO-12-042 and PI-18-183), and all participants provided their written informed consent. All of the research involving human research participants was performed in accordance with the Declaration of Helsinki.

### Proteomic analysis.

A custom-designed chip previously used in a study of Alzheimer’s disease (9) was used to detect and quantify 185 proteins in plasma samples. After normalization and clustering analyses, differences between groups of patients were analyzed using the *t* test, and molecules with a significance level (*P* value < 0.05 and FDR < 0.1) were included in further analyses. The functional analysis was performed using the application STRING: functional protein association networks (https://string-db.org).

### Proximity extension assay.

CSF samples were used for proximity extension analysis with Olink (https://www.olink.com/data-you-can-trust/technology/) for evaluation of neurology, neuroexploratory, and inflammation panels. Relative expression levels are expressed as normalized protein expression (NPX).

### Ultrasensitive single-molecule array.

Plasma samples were used for ultrasensitive single-molecule array (SIMOA)-based detection of tau, NFL, GFAP, and UCHL1 on an SR-X instrument (Quanterix). We used the commercially available Neuro 4-plex B kit for absolute quantification of tau, NFL, GFAP, and UCHL1. In brief, samples were thawed and centrifuged and then plated and diluted 4× with the sample dilution buffer to start the establish protocol for SIMOA. Antibody-attached beads designed to bind to specific targets were incubated with the samples, before secondary fluorescent antibodies were added. The plates were loaded into SIMOA array discs in which each well holds one bead and the enzymatic signal can be read.

### SIRT2 enzyme-linked immunosorbent assay.

Human SIRT2 enzyme-linked immunosorbent assay (ELISA) kit (Aviva Systems Biology) was used to measure the SIRT2 levels in plasma, and the absolute levels were quantified by applying a 4-parameter logistic curve analysis.

### Neuropsychological assessment.

Cognitive evaluation covered 6 cognitive domains to provide a global composite score (2 measures per domain, global NPZ-12). This included a digit test of the Wechsler adult intelligence scale (WAIS-IV); the trail making test (TMT-A) and the symbol digit modalities test (SDMT); grooved pegboard test (GPT); California verbal learning test (CVLT-II); the initial letter “p” and the animals test; and the trail making test (TMT-B) and the Tower of London test (TOL). The vocabulary test of the WAIS-IV was used to estimate premorbid intelligence.

### Brain image assessment.

Neurological image data were obtained by magnetic resonance imaging (MRI) (3 Tesla Magnetic Resonance Imaging Siemens Verio scanner). A high resolution T1-weighted three-dimensional (3-D) structural image using a 3-T scanner (Siemens Verio; Siemens Healthcare Sector, Germany) with a 32-phased-array head coil (192 slices in the axial plane; repetition time = 1,900 ms; echo time = 2.72 ms; flip angle = 9°; field of view = 260 × 260 mm; matrix size = 256 × 256 pixels; in-plane resolution = 0.96 × 0.96 mm^2^; slice thickness = 0.9 mm) was measured. After preprocessing and inspection for the presence of artifacts, all imaging time points were processed following a standard VBM-DARTEL pipeline to obtain MNI normalized and modulated images. Images were spatially smoothed with an 8-mm full width at half maximum (FWHM) isotropic Gaussian kernel. Differences at the whole-brain and voxel-wise level with a *P* < 0.05 significance threshold were explored. These analyses were controlled for age and total gray matter volume. Voxel values from significant regions were extracted to perform further statistical comparisons.

### Bioinformatic analysis of published transcriptomics.

The data set from the NCBI Gene Expression Omnibus (GEO) (http://www.ncbi.nlm.nih.gov/geo/) databank (accession number GSE28160) was used for evaluation of *SIRT2* expression levels in postmortem brain tissues (47).

### Real-time PCR.

RNA samples from PBMC dry pellets were retrotranscribed and TaqMan gene expression assay (Applied Biosystems) was used for detection of *SIRT2* (Hs01560289_m1) and *TBP* (Hs99999910_m1). Gene amplification was performed in an Applied Biosystems 7500 Fast real-time PCR system thermocycler, and the relative expression was calculated as 2^−Δ^*^CT^* (where *C_T_* is the median threshold cycle from 3 replicates).

### Determination of HIV proviral DNA.

HIV proviral DNA was quantified in PBMCs by droplet digital PCR (ddPCR) in duplicates as previously described (48). Briefly, two different primer/probe sets annealing to the 5′ long terminal repeat and Gag regions, respectively, were used to circumvent sequence mismatch in the patient proviruses, and the *RPP30* housekeeping gene was quantified in parallel to normalize sample input. Raw ddPCR data were analyzed using the QX100 droplet reader and QuantaSoft v.1.6 software (Bio-Rad).

### HIV replication of PHA blasts and monocyte-derived macrophages.

Isolated PBMCs from non-HIV-infected donors were stimulated with PHA (5 μg/mL) and interleukin-2 (IL-2) (10 U/mL). After 3 days, PHA blasts were infected with the HIV_NL4-3_ strain (multiplicity of Infection [MOI], 0.01). For monocyte-derived macrophages (MDMs), PBMCs were depleted using the EasySep human monocyte enrichment kit (Stem Cell). Monocytes were then incubated with macrophage colony-stimulating factor (M-CSF) at 1 μg/mL for 4 days before infection with the HIV_BaL_ strain (MOI, 0.01). HIV replication in PHA blasts and MDMs was evaluated under the following conditions: zidovudine (AZT) (200 μg/mL), dimethyl sulfoxide (DMSO), 10 μM AK-1 (3-[(hexahydro-1H-azepin-1-yl) sulfonyl]-N-(3-nitrophenyl)-benzamide). After 3 days or 1 week in PHA-blast and 4 days in MDM, p24 in supernatant was quantified by ELISA (INNOTEST HIV p24 antigen MAb).

### HIV replication of microglial cells.

Primary microglial cells (iCell Microglia; FUJIFILM Cellular Dynamics) were thawed and cultured for 3 days in a 96-well plate at 20,000 glial cells/well following the manufacturer’s recommendations. After 3 days, microglial cells were infected with the HIV_NLAD8_ strain (MOI, 0.01), and after 16 h, cells were washed. Infected microglial cells were then incubated in the absence or presence of 10 μM AK-1 (Sigma-Aldrich) or AZT (200 μg/mL). After 3 days, p24 in the culture supernatant was quantified by ELISA (INNOTEST HIV p24 antigen MAb).

### *In vitro* HIV reactivation.

The J-LAT A2 cells, which comprises transfected Jurkat cells with a green fluorescent protein (GFP)-encoding HIV minigenome (49), were stimulated with PMA (1 ng/mL) and cultured in the presence or absence of AK-1 (10 μM). After 24 h, GFP expression was evaluated on a fluorescence-activated cell sorter (FACS) Canto flow cytometer (Becton, Dickinson), and the data were analyzed using FlowJo version 10 software.

### Statistical analysis.

Mann-Whitney *U* test, Wilcoxon matched pairs tests and paired and unpaired *t* tests, and analysis of variance (ANOVA) test for multiple comparisons corrected by the original FDR method of Benjamini and Hochberg test were applied using GraphPad Prism, version 8. The Spearman's rank test was applied for correlation analyses. For all analyses, *P* values of <0.05 were considered statistically significant.
